# Health Services Usage in Patients Receiving Buprenorphine for Opioid Use Disorder or Long-Term Opioid Therapy for Chronic Pain: Retrospective Cohort Study

**DOI:** 10.2196/66596

**Published:** 2025-06-19

**Authors:** Samuel T Savitz, Maria A Stevens, Bidisha Nath, Gail D'Onofrio, Edward R Melnick, Molly M Jeffery

**Affiliations:** 1 Division of Health Care Delivery Research Kern Center for the Science of Health Care Delivery Mayo Clinic Rochester, MN United States; 2 Department of Emergency Medicine Yale School of Medicine Yale University New Haven, CT United States; 3 Department of Chronic Disease Epidemiology Yale School of Public Health Yale University New Haven, CT United States; 4 Department of Internal Medicine Yale School of Medicine Yale University New Haven, CT United States; 5 Department of Biostatistics Yale School of Public Health Yale University New Haven, CT United States; 6 Department of Emergency Medicine Mayo Clinic Rochester, MN United States

**Keywords:** telemedicine, opioids, buprenorphine, health care utilization, telehealth, utilization, usage, claims data, services, OUD, opioid use disorder, retrospective, cohort, pain, chronic

## Abstract

**Background:**

Patients using buprenorphine for opioid use disorder (OUD) or long-term opioid therapy for chronic pain are at risk for poor outcomes if care is interrupted. Both treatments are highly regulated, with prepandemic requirements for in-person care. COVID-19 may have resulted in barriers to accessing in-person care through disruptions in care delivery. However, there were also opportunities for improved access to telemedicine visits through policy changes.

**Objective:**

This study aims to evaluate changes in health care and telemedicine use during the COVID-19 pandemic among patients using buprenorphine for OUD and long-term opioid therapy for chronic pain.

**Methods:**

We used administrative claims data for commercially insured and Medicare Advantage patients from the OptumLabs Data Warehouse. We included patients using buprenorphine for OUD or long-term opioid therapy for chronic pain compared to patients with another chronic condition without similar prescribing restrictions: serious mental illness. We evaluated changes in in-person and telemedicine care by comparing rates of services by physician specialty, type of service, and the percentage of visits through telemedicine. Changes in usage were measured using a difference-in-differences approach with Poisson regression. The results are presented as incident rate ratios (IRR).

**Results:**

We found declines in in-person visits in April 2020 across the buprenorphine, chronic opioids, and serious mental illness cohorts. The largest declines were for specialties that rely on in-person treatment, such as emergency medicine (IRR range 0.60-0.62), orthopedics (IRR 0.48-0.52), cardiology (IRR 0.64-0.78), and oncology (IRR 0.77-0.81). In contrast, there were smaller declines for specialties that could more easily transition to telemedicine, namely family practice (IRR 0.80-0.92), mental health (IRR 0.92-1.01), and pain medicine (IRR 0.87-1.08). The percentage of telemedicine visits for these specialties ranged from 30% to 51% in the period. There were also large declines for specific services, including emergency medicine (IRR 0.53-0.89), physical therapy (IRR 0.24-0.72), and new office visits (IRR 0.38-0.64). By January 2022, usage was similar to prepandemic levels, but the percentage of telemedicine visits remained elevated for family practice (10%-14%), mental health (34%-43%), and pain medicine (11%-15%) through January 2022. The results were similar across the cohorts, although in April 2020 there was a modest decrease (IRR 0.87) for pain medicine in the serious mental illness cohort, but the differences were not significant for the buprenorphine (IRR 1.08) and chronic opioids (IRR: 0.99) cohorts.

**Conclusions:**

These findings highlight the value of telemedicine to maintain access among people at risk for poor outcomes if care is interrupted. While flexibilities in the regulation of telemedicine services that arose during the pandemic have been temporarily extended multiple times, they are set to expire in 2025 without further action. Making these changes to telemedicine regulation permanent may benefit vulnerable patient populations who face access to care challenges.

## Introduction

### Background

The COVID-19 pandemic caused disruptions to the delivery of health services [1-3]. During the early pandemic period, there were large decreases in care [2]. The decreases were most pronounced for services that could not easily transition to telemedicine such as emergency medicine, which relies heavily on physical examinations, laboratory tests, and imaging that must be done in person [3,4]. In contrast, declines tended to be smaller for services like mental health care that are less dependent on physical examinations and tests [3,5].

In response to the disruption in the delivery of health services, both public and private insurers loosened restrictions on telemedicine visits. Medicare expanded telemedicine coverage nationwide starting March 6, 2020, under an 1135 waiver [6]. The Centers for Medicare and Medicaid Services also allowed insurers to modify plan coverage midyear to provide greater coverage of telemedicine services [7]. The Coronavirus Aid, Relief, and Economic Security (CARES) Act in May 2020 provided additional flexibility for insurers with respect to telemedicine coverage [8]. In response, most private plans began offering telemedicine services with predeductible coverage [9].

There were also changes to the regulation of prescribing controlled substances. Starting on March 31, 2020, the Drug Enforcement Administration (DEA) allowed clinicians to prescribe controlled substances such as chronic opioid analgesic therapy and buprenorphine through telemedicine without first having an in-person visit [10]. In addition, the DEA began to relax the requirements for obtaining a Drug Addiction Treatment Act (2000) waiver, also known as the X-Waiver [11]. On April 27, 2021, the Department of Health and Human Services relaxed the training requirement for clinicians who were treating up to 30 patients with buprenorphine at a given time [11]. Then, the X-Waiver was eliminated on December 29, 2022, as part of the Consolidated Appropriations Act of 2023 [12].

### Buprenorphine for Opioid Use Disorder and Chronic Opioid Analgesic Therapy

Disruptions in access to care during the pandemic presented health risks to all patients [13]. In this context, 2 populations of particular concern are patients who use buprenorphine for opioid use disorder (OUD) and patients who use chronic opioid analgesic therapy. These patients need ongoing treatment, including monthly prescriptions, to manage chronic health problems and already faced access-to-care barriers before the pandemic [14]. These patients are at high risk for fatal and nonfatal opioid overdoses [15-19]. In the early months of the pandemic, there were increases in emergency usage related to opioids including emergency department (ED) visits for nonfatal opioid overdoses [20] and emergency medical service runs for fatal and nonfatal opioid-related concerns [21]. Disruption in access for patients who use buprenorphine for OUD and patients who use chronic opioid analgesic therapy may have contributed to these issues.

Patients receiving opioids for chronic pain or buprenorphine for OUD may be more likely to experience disruptions in care since buprenorphine and chronic opioid analgesic therapy are controlled substances with restrictions on prescribing [22-26]. Further, clinicians prescribing opioid analgesics face restrictions that vary by state and by payer, including the use of prescription drug monitoring programs, quantity limits, and duration limits [24,25,27]. Therefore, disruptions in care may affect continued access to buprenorphine or opioid therapy.

### Gap in the Literature

It is important to understand how pandemic-related care disruptions have affected these populations to inform future resource allocation and policy decisions related to ensuring ongoing access. Understanding the impact of the increase in access to telemedicine is also important, given that its continuation past the pandemic period is in question. While general population use of health care services and telemedicine has been reported [3], there is limited evidence on the experience among patients particularly vulnerable to access disruption.

### Study Overview

Our objective was to assess the change in the use of health services and the impact of telemedicine on access to care for 2 patient cohorts facing barriers to access. We used a large claims database to analyze trends in health services usage for patients who have fills for buprenorphine for OUD or chronic opioid analgesic therapy and compare them to patients with another chronic condition that does not generally have prescribing restrictions: serious mental illness (SMI). We selected SMI for comparison since SMI includes chronic conditions, but unlike buprenorphine and most opioid analgesics, most treatments for SMI can be written with refills and there are few or no requirements for in-person care. SMI is also a common comorbidity for patients with OUD or chronic pain [28-30]. The purpose of including SMI as a comparison group was to descriptively assess whether the observed trends were specific to patients with buprenorphine for OUD or chronic opioid analgesic therapy.

## Methods

### Data Source

We evaluated the use of services and access to care from January 2018 to February 2022, using data from the OptumLabs Data Warehouse (OLDW), a deidentified, national, medical claims database [31]. OLDW includes claims data for individuals with commercial and Medicare Advantage (MA) coverage. We report the study in accordance with the STROBE (Strengthening the Reporting of Observational Studies in Epidemiology) checklist for cohort studies (Multimedia Appendix 1) [32].

### Patients

We compared 3 cohorts of patients with chronic conditions that require ongoing treatment and may be affected by changes in access to care: patients with fills for buprenorphine formulations to treat OUD, patients with fills for chronic opioid analgesic therapy, and patients with SMI. We also evaluated heart failure as an additional nonmental health comparison condition and included results in Multimedia Appendix 2 as an additional sensitivity analysis. We separately identified patients belonging to each cohort. First, we identified patients with fills for buprenorphine to treat OUD. The definition for a buprenorphine episode is a period in which the patient never went more than 60 days without an active buprenorphine prescription. We only included buprenorphine formulations that were for treating OUD, excluding those used only to treat pain. We did not require patients to have a diagnosis for OUD to be included in the cohort. Second, we identified patients with fills for chronic opioid analgesic therapy. The definition of a chronic opioid episode is a period in which the patient never went more than 30 days without an active opioid prescription and had at least 10 fills or 120 days’ supply. We only included buprenorphine formulations that were for treating pain. Third, we identified patients who had at least one inpatient diagnosis or two outpatient diagnoses at least 1 day apart for SMI in the previous 6 months. SMI codes came from an Arizona block grant for serious emotional disturbance patients. Patients in the SMI cohort did not have to have received medication to be in the cohort. The cohorts were not mutually exclusive. Patients in all cohorts had to have at least 30 days of medical and pharmacy insurance enrollment in January 2018-February 2022 and 183 days of continuous medical and pharmacy coverage (see “Sample Flow Diagram” in Multimedia Appendix 3). To make the cohorts more comparable, we made the start date for the evaluation of the use of at least 60 days after the beginning of continuous enrollment. Censoring occurred upon the end of continuous enrollment, February 28, 2022, at the end of buprenorphine or chronic opioid episode, and when the patient no longer had at least one inpatient diagnosis or two outpatient diagnoses for SMI at least 1 day apart for the SMI cohort. During the study period, patients could enter and be removed from the analysis depending on whether they meet the inclusion and exclusion criteria on a given date.

### Measures

We first characterized the cohorts using descriptive statistics, including diagnoses for SMI [33], and selected Elixhauser comorbidities [34,35]. The key outcome was the use of health care services. We measured in-person and telemedicine visits as the specialty of the provider on the claim and the Berenson-Eggers Types of Service Codes (BETOS) 2.0, which categorizes procedure codes into meaningful groups for the management of clinical conditions [36]. Previous work has analyzed usage patterns using the provider specialty field in OLDW [3]. We defined telemedicine usage as claims with either a procedure modifier of GT, GQ, or 95 or a Current Procedural Terminology (CPT) code of 99441, 99442, 99443, G0425, G0426, G0427, G0406, G0407, G0408, or G045 [6]. We measured usage both overall among the 3 cohorts and broken out by insurance categories (commercial insurance [COM], MA younger than 65 y [MA <65 y], and MA aged 65 y or older [MA 65+]). People younger than 65 years qualify for MA due to long-term disability or certain serious medical conditions, including end-stage renal disease and experience different patterns in health care usage [37]. The numerator was the number of person-days of service, and the denominator was the number of enrollee months in the period.

We initially focused on commonly accessed clinician specialties that are important for these patient populations, selecting a range of specialties that differed in how readily they could be performed through telemedicine. The specialties initially considered were cardiology, emergency medicine, family practice, mental health, oncology, orthopedics, and pain medicine. The mental health category included visits with clinicians who have a specialty in psychiatry, psychology, or social work. The pain medicine category included visits with a specialty of anesthesiology for which the claim had an evaluation and management code. After exploring these specialties, we narrowed the focus to family practice, mental health, and pain medicine. We selected these specialties as particularly conducive to telemedicine visits since they are generally not procedure-based and often focus on the management of chronic conditions. Pharmacotherapy can be prescribed through telemedicine, and it is the most common treatment modality for mental health conditions like depression [5] and is one of the most common treatment modalities for pain medicine [38]. Many of the most common reasons for primary care visits include conditions that could be managed, in part, through telemedicine, including hypertension, diabetes, depression, and anxiety [39]. Further, these specialties are especially important for the study populations since clinicians in these specialties are among the most likely to prescribe buprenorphine to treat opioid use disorder [40]. Results for the other specialties are available in Multimedia Appendices 4 and 5.

We also evaluated 3 BETOS procedure categories: emergency medicine, physical therapy, and new office visits. These categories represent services that may be important for the included patient cohorts and for which there may have been reduced access because of their reliance on in-person visits. Emergency medicine often requires physical examinations and tests [4], as well as time-sensitive physical interventions, including, for example, administering naloxone to patients presenting with an overdose [20]. Similarly, physical therapy relies on physical examinations and treatments [41] and is an important nonpharmacological approach to pain management [42]. New office visits may be particularly important for patients who no longer had an established provider for managing prescriptions or initiated buprenorphine in the emergency department after an overdose [43].

We also assessed how the pandemic-associated changes in usage have affected access to care for mental health. We defined mental health access in each month as the percentage of patients with a psychiatry or behavioral health visit in the 6 months before that month. We measured access among the subset of patients in the buprenorphine and chronic opioid cohorts who also had SMI. The purpose of this measure was to assess whether patients with chronic mental health condition were receiving regular follow-up care.

### Design

For usage outcomes, we calculated the rate of person-days of service per 100,000 enrollee-months. To assess whether there were significant differences in usage, we performed an analysis using a difference-in-differences (DiD) approach. This approach evaluates the difference before and after COVID-19 and accounts for temporal patterns in usage by month. While DiD is often used for causal inference [44,45], in this study, we use the DiD approach to descriptively assess whether usage trends changed over time differentially for one group versus another. We focused on four comparisons to January 2020 as the reference: April 2020, July 2020, July 2021, and January 2022. We selected the 4 time points to represent key periods during the pandemic: early pandemic, April 2020; second wave, July 2020; Delta wave, July 2021; and Omicron wave, January 2022. For example, usage changes in April 2020 were calculated by evaluating whether the change in usage between January and April 2020 was different from that between January and April 2019. We chose the DiD approach for 2 reasons: first, there are no clear-cut points to define the major waves of the pandemic, which shifted somewhat gradually over time and hit at different times across the country. Second, we wanted to compare the changes with respect to a prepandemic baseline. Using our approach, we were able to compare the 4 time points to the reference time point of January 2020 to see if usage was lower or higher than that before the pandemic. We implemented the analysis using Poisson regression with the use rate as the outcome. The models also included an offset term for the natural log of the number of eligible enrollees in each cohort to model the number of events as a rate of events per enrollee [46]. We present the results as incident rate ratios (IRRs) with 95%. All analyses were performed using Stata (version 16; StataCorp, 2019).

### Ethical Considerations

The study was submitted to the Mayo Clinic institutional review board (application ID 25-003376) and deemed exempt from institutional board review under Exemption Category 4 (secondary research of existing data). The data for this study were deidentified to ensure privacy for sensitive health care information.

## Results

### Descriptive Statistics

We identified 69,032 patients in the buprenorphine cohort, 817,381 in the chronic opioid use cohort, and 4,247,090 in the SMI cohort. The cohorts ranged (Table 1) from 32% (4426/13,717) female (COM buprenorphine cohort) to 72% (223,546/312,291) female (MA 65+ SMI cohort). The buprenorphine cohort was more likely to meet the Elixhauser comorbidity drug use disorder definition [53% (7217/13,717) COM compared to <10% for other cohort] and less likely to be female.

**Table 1:** Descriptive statistics for buprenorphine, chronic opioids, and serious mental illness cohorts as of January 2020.

Variable		Commercial			Medicare Advantage <65 years							Medicare Advantage 65+ years				
		Buprenorphine	CO^b^	SMI^c^	Buprenorphine		CO	SMI		Buprenorphine			CO		SMI	
Total (N)		13,717	69,979	534,318	5059	92,432			118,077		2506			180,900		312,291
**Age range (years), % (n)**																
	0-25	3.2 (440)	0.5 (344)	24.6 (131,297)	8.0 (404)		2.3 (2083)	6.9 (8133)		—^d^			—		—	
	0-39															
	26-39	43.5 (5967)	10.2 (7149)	30.3 (161,984)	92.0 (4655)		97.8 (90,349)	93.1 (109,944)		—			—		—	
	40-64	51.7 (7091)	79.7 (55,764)	41.4 (221,202)	—		—	—		87.3 (2188)			58.0 (104,904)		50.2 (156,638)	
	65+	1.6 (219)	9.6 (6722)	3.7 (19,835)	—		—	—		12.7 (318)			42.0 (75,996)		49.8 (155,653)	
	65-74	—	—	—	—		—	—		87.3 (2188)			58.0 (104,904)		50.2 (156,638)	
	75+	—	—	—	—		—	—		12.7 (318)			42.0 (75,996)		49.8 (155,653)	
**Gender, % (n)**																
	Women	32.3 (4426)	57.0 (39,904)	65.0 (346,965)	49.4 (2500)		59.4 (54,939)	63.2 (74,578)		43.4 (1088)			64.1 (115,889)		71.6 (223,546)	
**Race and ethnicity, % (n)**																
	Asian or PI^e^	0.9 (129)	1.1 (764)	2.2 (11,991)	0.6 (30)		0.7 (648)	1.0 (1145)		0.5^f^ (12)			0.9 (1557)		1.3 (4064)	
	Black	5.7 (781)	8.4 (5872)	6.4 (33,959)	12.0 (607)		21.7 (20,046)	18.5 (21,840)		13.7 (343)			17.3 (31,373)		11.5 (35,813)	
	Hispanic	5.1 (705)	6.7 (4713)	7.7 (41,075)	5.5 (276)		6.4 (5865)	8.2 (9708)		5.3 (132)			6.0 (10,889)		9.3 (28,990)	
	White	70.3 (9644)	71.5 (50,066)	66.7 (356,143)	69.1 (3494)		59.8 (55,299)	59.1 (69,760)		69.4 (117,989)			65.2 (117,989)		65.7 (205,273)	
	Other	17.9 (2458)	12.2 (8564)	17.1 (91,150)	12.9 (652)		11.4 (10,574)	13.2 (15,624)		11.2 (281)			10.6 (19,092)		12.2 (38,151)	
**Elixhauser comorbidities, % (n)**																
	Alcohol use disorder	3.8 (524)	0.9 (659)	3.0 (16,247)	6.8 (345)		1.9 (1780)	5.1 (6072)		5.5 (137)			1.1 (1905)		2.4 (7351)	
	Drug use disorder	52.6 (7217)	4.3 (2998)	3.3 (17,655)	57.4 (2904)		7.5 (6973)	9.6 (11,288)		50.2 (1259)			4.0 (7287)		2.5 (7772)	
	Diabetes	2.8 (384)	11.0 (7713)	4.8 (25,686)	14.8 (746)		29.6 (27,327)	27.6 (32,540)		18.4 (462)			30.7 (55,500)		28.6 (89,165)	
**Other comorbidities, % (n)**																
	SMI^g^	25.7 (3526)	19.3 (13,528)	100 (534,318)	50.2 (2537)		34.5 (31,907)	100 (118,077)		38.9 (974)			23.5 (42,452)		100 (312,291)	
	Anxiety disorders	17.1 (2348)	12.8 (8976)	62.4 (333,431)	30.7 (1552)		20.5 (18,976)	42.9 (50,604)		22.8 (571)			13.6 (24,650)		44.2 (138,104)	
	Bipolar disorders	2.7 (376)	1.5 (1055)	6.7 (35,969)	13.4 (678)		5.7 (5243)	20.9 (24,676)		3.4 (84)			1.5 (2780)		5.9 (18,289)	
	Depressive disorders	14.4 (1977)	10.8 (7543)	50.1 (267,522)	30.0 (1518)		21.3 (19,961)	50.0 (59,066)		25.8 (27,036)			15.0 (27,036)		52.6 (164,331)	
	Dissociative disorders	0.0 (0)^f^	0.0 (0)^f^	0.0 (188)	0.1 (0)^f^		0.0 (18)	0.1 (123)		0.0 (0)^f^			0.0 (0)^f^		0.0 (70)	
	Obsessive-compulsive disorders	0.4 (53)	0.2 (117)	2.6 (11,653)	0.6 (28)		0.3 (241)	1.3 (1529)		0.2 (0)^f^			0.1 (180)		0.6 (1898)	
	Other mood disorders	0.8 (104)	0.3 (173)	1.7 (9321)	1.3 (67)		0.5 (442)	1.7 (1966)		1.0 (26)			0.3 (536)		1.2 (3841)	
	Personality disorders	0.3 (47)	0.1 (100)	0.9 (5049)	1.7 (88)		0.6 (571)	2.7 (3126)		0.4 (0)^f^			0.2 (275)		0.5 (1616)	
	Psychotic disorders	0.5 (65)	0.2 (121)	1.4 (7640)	4.2 (212)		2.2 (2054)	16.1 (18,977)		1.5 (38)			1.0 (1774)		5.7 (17,715)	
	Trauma-related disorders	2.4 (334)	1.3 (909)	5.9 (31,661)	8.6 (437)		3.4 (3150)	10.5 (12,345)		2.6 (66)			0.7 (1246)		2.2 (6699)	

^b^CO: chronic opioid.

^c^SMI: serious mental illness.

^d^Not applicable.

^e^PI: Pacific Islander.

^f^ Number suppressed due to a cell size <11

^g^The number of Asian or PI patients was <11 for the Medicare Advantage 65+ buprenorphine cohort, so we combined this group with the Other/Unknown group.

### Specialty Usage

**Figure 1:**
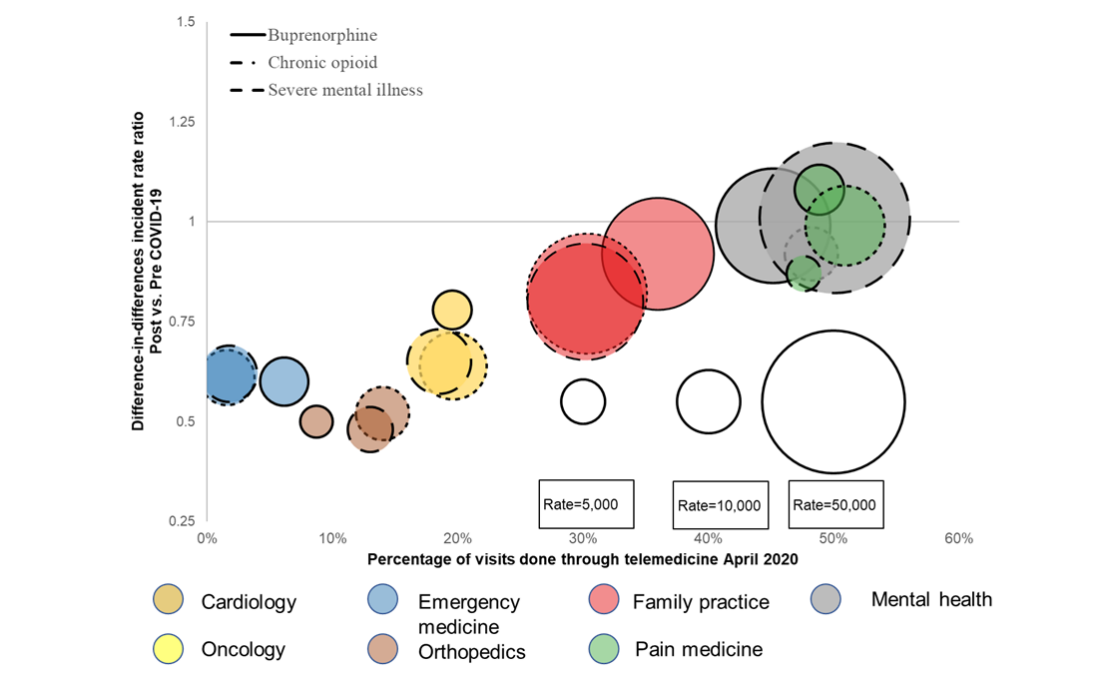
Difference-in-differences incidence rate ratio for change in utilization and telemedicine use across seven specialty categories in the buprenorphine, chronic opioids, and serious mental illness cohorts pre versus post COVID-19 April 2020.

In April 2020, the IRR for changes in use and the percentage of telemedicine visits, compared to those before COVID-19, were similar for patients in the buprenorphine and chronic opioid cohorts relative to the SMI cohort across 7 key specialties (Figure 1; Multimedia Appendix 4). Usage declined relative to prepandemic for emergency medicine (IRR range 0.60-0.62), orthopedics (IRR 0.48-0.52), cardiology (IRR 0.64-0.78), and oncology (IRR 0.77-0.81), while the corresponding percentage of telemedicine visits for these services remained below 20%. In contrast, usage was similar during COVID-19 compared to that before COVID-19 for family practice (IRR 0.80-0.92), mental health (IRR 0.92-1.01), and pain medicine (IRR 0.87-1.08). The use of telemedicine visits was also greater than before the pandemic and ranged from 30% to 51%.

While the results were generally similar for the buprenorphine and chronic opioid cohorts compared to the SMI cohort, some differences were present. For pain medicine, the buprenorphine (IRR 1.08) and chronic opioid (IRR 0.99) cohorts experienced no significant difference in usage, whereas the SMI (IRR 0.87) cohort experienced a modest decrease compared to each cohort’s usage before the pandemic.

In Figure 1, bubble size is the rate of person-days of service per 100,000 patient-months in April 2020. The incidence rate ratio for oncology in the buprenorphine cohort is not displayed because the rate is below 1000 person-days of service per 100,000 person-months.

**Figure 2:**
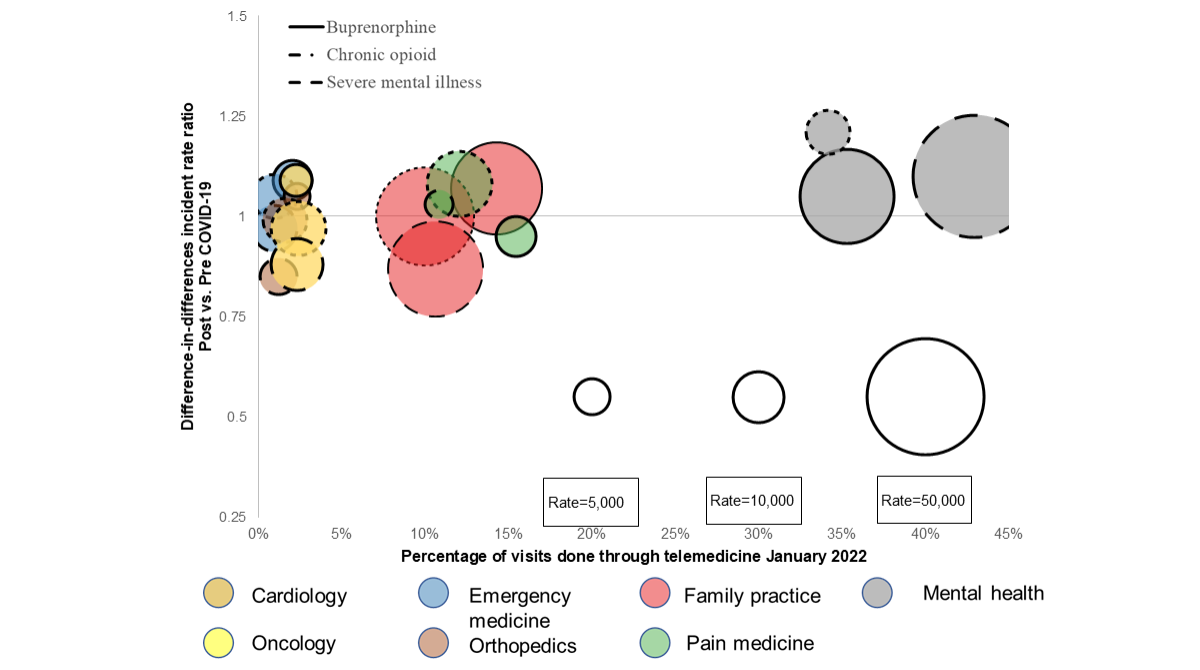
Difference-in-differences incidence rate ratio for change in utilization and telemedicine use across seven specialty categories in the buprenorphine, chronic opioids, and serious mental illness cohorts pre versus post COVID-19 January 2022.

In January 2022 during the Omicron wave, usage was similar to prepandemic levels (Figure 2; Multimedia Appendix 4). Telemedicine use was also much lower than in April 2020. The percentage of visits that were telemedicine was 5% or lower for all cohorts of emergency medicine, oncology, orthopedics, and cardiology. In contrast, telemedicine use remained moderate for family practice (10%-14% of visits) and pain medicine (11%-15%) and high for mental health (34%-43%).

Separating the cohorts by insurance categories revealed similar patterns (Table 2, Multimedia Appendix 4). Usage was lower for family practice (IRR 0.74-0.95 for COM, 0.84-0.87 for MA <65 y, and 0.79-0.93 for MA 65+) in April 2020 than in April 2019 (Table 2). However, there was a smaller reduction or no decrease for pain medicine (IRR 0.84-0.96 for COM, 0.97-1.13 for MA <65 y, and 0.79-1.12 for MA 65+) and mental health (IRR 0.92-1.03 for COM, 1.00-1.08 for MA <65 y, and 0.80-1.05 for MA 65+). The percentage of telemedicine visits for April 2020 was high for all specialties across all cohorts and insurance groups. Telemedicine ranged from 23% to 47% of visits for family practice, 45%-55% for pain medicine, and 40%-56% for mental health. In contrast, the percentage of telemedicine visits was below 2% for all cohorts in April 2019.

**Table 2:** Rate per 100,000 person-months, difference-in-differences incidence rate ratios, and telemedicine use for select specialties in the buprenorphine, chronic opioids, and serious mental illness cohorts April 2020-January 2022. The rate is in terms of person-days of service per 100,000 patient-months.

Insurance (cohort)				January 2020, rate per 100,000 PMs^a^	April 2020						July 2020					July 2021					January 2022		
					IRR^b^		Tel (%)^c^			IRR		Tel (%)			IRR		Tel (%)			IRR			Tel (%)
**Family practice**																							
	**Commercial**																						
		Bup^d^	23,467.1			0.95		35	1.03				20	0.90^e^				11	0.90^e^			15	
		CO^f^	21,461.9			0.84^g^		42	0.96^h^				19	0.93^i^				8	1.00			12	
		SMI^i^	16,491.2			0.74^g^		47	1.00				23	0.89^g^				10	0.98^e^			15	
	**MA^j^ <65 y**																						
		Bup	44,592.4			0.84^g^		37	0.93				20	0.94				11	1.01			15	
		CO	38,879.2			0.87^g^		31	0.99				14	0.98				6	1.00			12	
		SMI	47,487.2			0.85^g^		30	0.99				15	0.87^g^				8	0.87^g^			12	
	**MA 65+**																						
		Bup	42,316.4			0.93		36	0.86^h^				18	1.01				9	1.19^h^			12	
		CO	38,328.8			0.79^g^		27	0.95^g^				12	0.93^g^				5	0.97^g^			9	
		SMI	53,511.9			0.79^g^		23	0.95^g^				12	0.79^g^				5	0.80^g^			8	
**Pain medicine**																							
	**Commercial**																						
		Bup	3886.0			0.95		45	0.98				34	0.91				10	0.71^g^			13	
		CO	16,513.7			0.96		55	0.99				31	0.97				11	0.92^g^			14	
		SMI	1310.1			0.84^g^		51	1.02				25	0.91^g^				9	0.88^g^			13	
	**MA <65 y**																						
		Bup	10,583.7			1.13		51	1.03				35	0.96				13	0.78^h^			16	
		CO	20,565.7			1.05^e^		47	1.08^g^				23	1.05^g^				9	0.97^h^			12	
		SMI	8394.9			0.97		48	1.11^g^				22	1.03				9	0.91^g^			12	
	**MA 65+**																						
		Bup	10,655.2			1.12		50	1.18				24	1.22				12	1.44^h^			16	
		CO	13,132.7			0.95^g^		52	1.03^h^				26	1.15^g^				9	1.17^g^			12	
		SMI	4057.6			0.79^g^		45	1.04				20	1.12^g^				7	1.12^g^			9	
**Mental health**																							
	**Commercial**																						
		Bup	31,061.2			0.97		41	1.11^e^				38	1.01				31	0.89^e^			33	
		CO	7047.7			0.92^e^		56	1.05				50	1.02				44	1.08^h^			46	
		SMI	72,180.4			1.03^g^		52	1.18^g^				51	1.08^g^				45	1.08^g^			48	
	**MA <65 y**																						
		Bup	39,397.7			1.00		50	1.06				48	1.15^e^				33	1.12^f^			36	
		CO	9484.2			1.06^e^		52	1.12^g^				47	1.18^g^				37	1.32^g^			40	
		SMI	51,595.6			1.08^g^		48	1.14^g^				43	1.09^g^				34	1.19^g^			37	
	**MA 65+**																						
		Bup	26,536.6			1.05		54	1.22^h^				48	1.38^g^				38	1.04			41	
		CO	6033.9			0.80^g^		40	0.89^g^				37	0.98				22	1.11^g^			25	
		SMI	30,906.6			0.91^g^		42	1.14^g^				35	1.01				28	1.10^g^			31	

^a^PM: person-month.

^b^IRR: incident rate ratio.

^c^Tel: telemedicine.

^d^Bup: Buprenorphine.

^e^*P*<.01.

^f^CO: chronic opioid.

^g^*P*<.001.

^h^*P*<.05.

^i^SMI: serious mental illness.

^j^MA: Medicare Advantage.

In Figure 2, bubble size is the rate of person-days of service per 100,000 patient-months in January 2022. The IRR for oncology in the buprenorphine cohort is not displayed because the rate is below 1000 person-days of service per 100,000 person-months.

Total usage for the same specialties largely recovered in July 2020 compared to July 2019 and remained at similar levels in July 2021 and January 2022. The percentage of telemedicine visits was lower for family practice and pain medicine, but not mental health (July 2021: 35%-51%; July 2021: 22%-45%; January 2022: 25%-48%).

### BETOS Usage

Using the BETOS classification, there were large declines in total usage in emergency medicine (IRR 0.53-0.89), physical therapy (IRR 0.24-0.72), and new office visits (IRR 0.38-0.64) in April 2020 compared to April 2019 (Table 3). Except for the buprenorphine cohort in the MA 65+ group for emergency medicine and physical therapy, usage significantly decreased for all cohorts and insurance groups. The declines were consistent across insurance groups. Usage mostly recovered by July 2020 for physical therapy and new office visits. However, usage was still moderately lower for emergency medicine for 10 out of 12 cohorts and insurance groups in July 2020. By July 2021, use of emergency medicine had further recovered. In January 2022, there was a mix of increases and decreases with no consistent pattern.

**Table 3:** Rate and difference-in-differences incidence rate ratios for emergency medicine, physical therapy, and new office visits Berenson-Eggers Types of Service Codes categories in the buprenorphine, chronic opioids, and serious mental illness cohorts April 2020-January 2022.

Insurance (cohort)				January 2020, rate		April 2020, DiD^a^		July 2020, DiD		July 2021, DiD		January 2022, DiD
**Emergency medicine**												
	**COM^b^**											
		Bup^c^	3228		0.53^d^		0.91		1.10		1.33^e^	
		CO^f^	5108		0.57^d^		0.79^d^		0.95		1.14^d^	
		SMI^g^	3994		0.54^d^		0.93^d^		0.93^d^		0.89^d^	
	**MA^h^ <65 y**											
		Bup	8545		0.60^d^		0.79^e^		0.86		1.25	
		CO	9614		0.58^d^		0.80^d^		1.08^d^		1.06^e^	
		SMI	15,770		0.63^d^		0.85^d^		0.92^d^		0.88^d^	
	**MA 65+**											
		Bup	6495		0.89		1.39^e^		1.67^i^		0.83	
		CO	6637		0.57^d^		0.87^d^		1.25^d^		1.16^d^	
		SMI	9567		0.60^d^		0.84^d^		1.01		1.02	
**Physical therapy**												
	**COM**											
		Bup	3228		0.63^d^		1.04		1.05		0.67^d^	
		CO	14,109		0.49^d^		0.86^d^		1.05^e^		1.02	
		SMI	13,228		0.44^d^		0.87^d^		0.92^d^		0.79^d^	
	**MA<65 y**											
		Bup	5243		0.24^d^		0.98		0.96		1.53^e^	
		CO	13,176		0.50^d^		0.99		1.14^d^		0.81^d^	
		SMI	22,090		0.62^d^		0.98		0.96^i^		0.72^d^	
	**MA 65+**											
		Bup	11,771		0.72		1.18		0.95		0.98	
		CO	21,934		0.64^d^		0.95^d^		1.16^d^		1.02	
		SMI	46,280		0.71^d^		1.02^i^		0.98^d^		0.84^d^	
**New office visits**												
	**COM**											
		Bup	6349		0.64^d^		1.05		1.04		1.27^i^	
		CO	8779		0.55^d^		0.90^d^		1.02		1.07^e^	
		SMI	9345		0.51^d^		1.02		0.97^i^		1.00	
	**MA<65 y**											
		Bup	8812		0.60^d^		1.08		0.98		0.75^e^	
		CO	8689		0.51^d^		0.95^e^		1.05		0.97	
		SMI	10,857		0.49^d^		0.97		0.96^e^		0.85^d^	
	**MA 65+**											
		Bup	9590		0.51^d^		0.82		1.08		1.03	
		CO	7444		0.41^d^		0.90^d^		1.16^d^		1.14^d^	
		SMI	9744		0.38^d^		0.92^d^		1.04^i^		0.94^d^	

^a^DiD: difference-in-differences.

^b^COM: Commercial insurance.

^c^Bup: Buprenorphine.

^d^*P*<.001.

^e^*P*<.05.

^f^CO: chronic opioid.

^g^SMI: serious mental illness.

^h^MA: Medicare Advantage.

^i^*P*<.01.

### Mental Health Access

**Figure 3:**
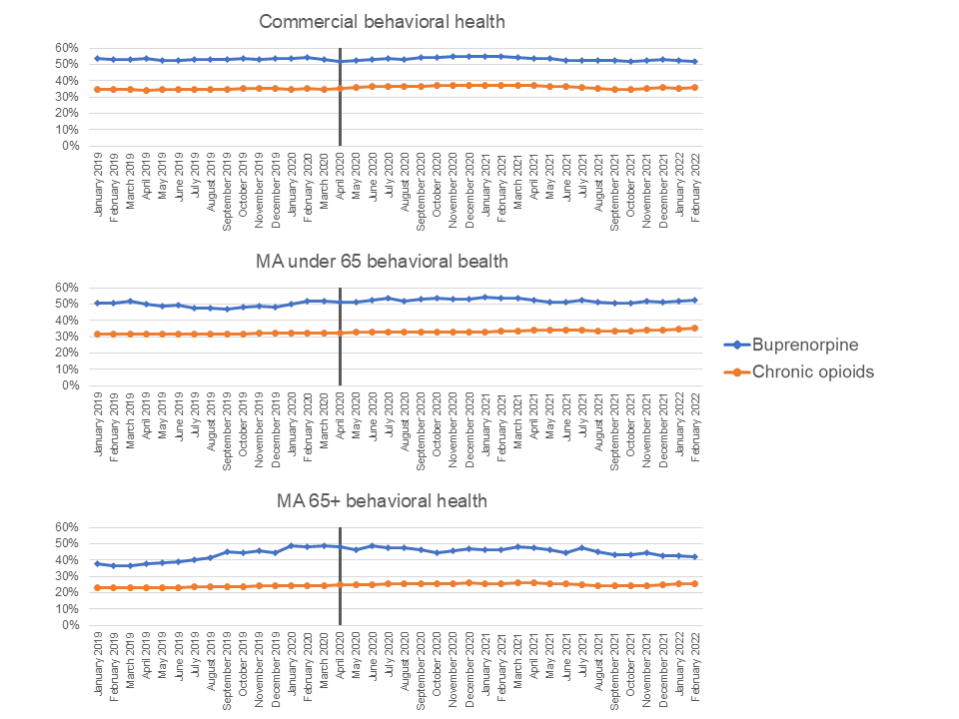
Percent of patients with behavioral health services in previous six months in the buprenorphine and chronic opioids cohorts January 2019-February 2022. The line indicates the beginning of the COVID-19 pandemic in April 2020. MA: Medicare Advantage.

Among patients in the buprenorphine and chronic opioid cohorts who also had a diagnosis for SMI, there were mixed results for access to mental health services. The percentage of patients with behavioral health visits in the previous 6 months fell slightly or stayed flat during the early COVID-19 period (Figure 3). The change from January 2020 to January 2022 ranged from a decrease of 4% points to an increase of 5% points. Patients in the buprenorphine cohort had decreases (–1% to –3%) whereas patients in the chronic opioid analgesic therapy cohort (2%-5%) had increases. The results were largely similar when evaluating psychiatry visits (Multimedia Appendix 5).

In Figure 3, The line indicates the beginning of the COVID-19 pandemic in April 2020. The outcome measures the percentage of patients with a visit in the usage category within the prior 6 months conditional on having coverage for at least 6 months.

## Discussion

### Summary

We found that among patients with fills for buprenorphine for OUD or chronic opioid analgesic therapy during the early period of COVID-19, the total use rate for many key specialties was similar or only slightly lower than prepandemic. This pattern appeared to be supported by the substitution of in-person visits with telemedicine. In addition, the relative usage and access for patients who use buprenorphine or chronic opioid analgesic therapy were similar or higher than another comparative chronic condition cohort, suggesting that these cohorts were able to maintain access to care during the pandemic.

We also found an expected substantial decrease in usage for specialties and services that could not be readily performed via telemedicine. While usage had largely recovered to prepandemic levels by the end of the study period, telemedicine use remained elevated, especially for mental health services. However, despite the continued use of telemedicine in mental health care, access did not improve during the study period—the availability of telemedicine appears primarily to have substituted for in-person visits rather than increasing overall access to mental health care. This may reflect the shortages in the mental health care workforce prevalent across much of the country [47].

### Services That Could Not Easily Shift to Telemedicine

We found decreased usage in April 2020 for service categories that could not easily transition to telemedicine, including emergency medicine, orthopedics, and physical therapy. These services are critical for many patients among the buprenorphine and chronic opioid analgesic cohorts, and the reduced access may have contributed to worse outcomes. Patients with overdoses may be seen in the ED, with about 21% of patients who have an opioid-related condition or opioid overdose as the primary diagnosis for the ED visit being admitted to the hospital [48]. One concern is that the decreased use of emergency medicine may include delayed care for opioid overdoses similar to what was found for myocardial infarction [49]. Instead, the rate of opioid overdose ED visits actually increased despite a decrease in all-cause ED visits [20]. However, the number of excess deaths from drug overdoses also increased during the early months of COVID-19 [50], and there may have also been more patients experiencing overdoses that did not reach the hospital. In addition, some patients who receive treatment for OUD have it initiated at the ED, so it is possible that the decline in access to the ED may have reduced opportunities to initiate treatment. This pattern may be related to the lower initiation of medications for OUD during the early months of the pandemic [51].

The decrease in usage for physical therapy is also concerning because chronic opioid analgesic patients who receive early physical therapy tend to have shorter durations and lower intensity of opioid use [52]. More research is needed to understand how the declines in access to emergency medicine, orthopedics, and physical therapy potentially affected buprenorphine and chronic opioid analgesic use during this period and whether there is an association with the increase in fatal opioid overdoses [53].

### Role of Telemedicine for Maintaining Access

These findings also suggest that the expanded use of telemedicine was important for maintaining access to certain specialties. Our finding that greater use of telemedicine was associated with a lower decline in usage was similar to another study focused on all patients [3]. Our study confirms that vulnerable patient populations, including those who receive buprenorphine or chronic opioid analgesic therapy as well as patients with SMI, depended on telemedicine for continued access to care. We also confirmed that usage largely recovered after the initial months of the pandemic. Telemedicine use declined for all specialties but remained higher than prepandemic for family practice and pain medicine and much higher for mental health. Despite the increased use of telemedicine for mental health, access to mental health services was largely unchanged.

Policy makers should consider our findings with respect to the role of telemedicine in maintaining access to key specialties when deciding regulations, including recent proposed changes from the DEA [54,55]. Continued access to telemedicine may be important for maintaining access to care during other disruptions in care delivery such as ongoing provider shortages and the need for OUD treatment given the high numbers of fatal opioid overdoses [53]. Telemedicine also has the potential to reduce disparities for patients in rural areas with limited access to mental health providers [56-59]. However, there are also inequities in broadband availability [60]. and digital literacy [61] that may affect access to telemedicine. Individuals needing OUD treatment tend to have lower income, with 7.5% of those with income <100% of the federal poverty line needing OUD treatment compared to only 2.5% of those with income ≥200% of the federal poverty line. Further, individuals needing OUD treatment are also more likely to live in counties outside of metropolitan areas (4.7% vs 3.5%) [62]. As such, difficulty accessing broadband may be particularly salient for many individuals needing OUD treatment. Other access barriers, such as shortages of mental health providers [63], and inequities in who uses telemedicine [64], may also limit improvements in access to telemedicine. Improvements in access may depend on telemedicine being available with minimal restrictions by encounter type and for both new and established patients and on policies being designed with consideration of equity principles [65].

However, access to telemedicine may worsen if temporary waivers of telemedicine regulation made during the pandemic are allowed to lapse. The DEA has continued to grant temporary extensions for permitting the prescribing of controlled medications for OUD such as buprenorphine through telemedicine. Most recently, the DEA extended the flexibility for the third time through December 31, 2025 [66]. However, since the extensions are temporary, the potential remains for this flexibility to no longer be extended in the future. Similarly, the CARES Act included telemedicine waivers that (1) allowed patients to reside in any part of the country and to be in any setting, (2) eliminated the need for an in-person visit before receiving behavioral health visits through telemedicine, (3) extended which practitioners were eligible to bill for telemedicine services, and (4) allowed audio-only telemedicine for some services [8,67]. These waivers were extended in the Consolidated Appropriations Act of 2023 and were set to expire on December 31, 2024 [12]. Then, the American Relief Act was passed in December 2024, and it temporarily extended the waivers until March 31, 2025 [68]. Instead of additional temporary extensions for the prescribing of buprenorphine through telemedicine and the general telemedicine waivers, a permanent revision to the regulations would help ensure continued access.

### Limitations

A limitation of this analysis is that the OLDW does not include uninsured individuals or those with Medicaid coverage, who are more likely to have challenges in access to care such as a lower rate of physicians accepting new patients with Medicaid compared to new patients with commercial or Medicare coverage [69]. Medicaid accounts for approximately 25% of spending for mental health treatment and 21% of spending for substance use disorder treatment [70]. As such, it is an important population, and more research is needed to understand how access and usage changed during the period for individuals with Medicaid coverage as well as individuals who were uninsured. While Medicaid is a major payer for substance use disorders, commercial insurance is also an important payer, covering 58% of nonolder adults with substance use disorder [71]. In addition, it is important to note that this study’s focus on people with health insurance allows us to assess the impact of the pandemic and increased coverage of telemedicine on people with important chronic healthcare needs, while abstracting away from access challenges related to insurance status.

### Conclusions

Among commercially insured and MA patients, the availability of telemedicine in the early period of the pandemic may have helped patients, including those who use buprenorphine and chronic opioid analgesic therapy, retain access to needed services. However, the continued use of telemedicine in mental health care did not improve access to care. For services among which access decreased, the decline was no greater for patients who use buprenorphine or chronic opioid analgesic therapy compared to patients with SMI. It will remain important to maintain access for these vulnerable populations in future disruptions to health care delivery.

## Appendix

Multimedia Appendix 1 STROBE (Strengthening the Reporting of Observational Studies in Epidemiology) checklist for cohort studies.

Multimedia Appendix 2 Heart failure cohort sensitivity analysis.

Multimedia Appendix 3 Sample flow diagram.

Multimedia Appendix 4 Additional results for specialty usage in the buprenorphine and chronic opioids cohorts, April 2020-January 2022.

Multimedia Appendix 5 Access to psychiatry visits in the buprenorphine and chronic opioids, cohorts, April 2020-January 2022.

## Abbreviations

BETOS: Berenson-Eggers Types of Service Codes
CARES: Coronavirus Aid, Relief, and Economic Security
CPT: Current Procedural Terminology
DEA: Drug Enforcement Agency
DiD: difference-in-differences
ED: emergency department
IRR: incident rate ratio
MA: Medicare Advantage
OLDW: OptumLabs Data Warehouse
OUD: opioid use disorder
SMI: serious mental illness
STROBE: Strengthening the Reporting of Observational Studies in Epidemiology

## Data Availability

The datasets generated or analyzed during this study are not publicly available because they were used under license for the current study in an agreement with OLDW. Data are, however, available from the authors upon reasonable request and with permission of OLDW.
