# Supplementary material for: Health Services Usage in Patients Receiving Buprenorphine for Opioid Use Disorder or Long-Term Opioid Therapy for Chronic Pain: Retrospective Cohort Study
Source: JMIR Form Res. 2025 Jun 19;9:e66596. doi: 10.2196/66596 (PMC12226777; doi:10.2196/66596)
Supplement: Multimedia Appendix 2 [file formative_v9i1e66596_app2.docx]

## Appendix 1: Heart Failure Cohort Sensitivity Analysis

Table 1: Descriptive Statistics for Heart Failure Cohort as of January 2020

| Variable | Level | Com | Level | MA <65 | MA 65+ |
| --- | --- | --- | --- | --- | --- |
| N |  | 38,482 |  | 33,588 | 254,702 |
| Age | 0-25 | 1.7 | 0-39 | 1.5 | - |
|  | 26-39 | 5.1 | 40-64 | 98.5 | - |
|  | 40-64 | 62.3 | 65-74 | - | 34.8 |
|  | 65+ | 30.9 | 75+ | - | 65.2 |
| Gender | Female | 36.0 | Female | 48.2 | 53.2 |
| Race/  Ethnicity | Asian/PI | 2.2 | Asian/PI | 1.0 | 1.7 |
|  | Black | 13.3 | Black | 29.5 | 17.9 |
|  | Hispanic | 7.6 | Hispanic | 7.1 | 8.9 |
|  | White | 63.7 | White | 49.0 | 60.2 |
|  | Other | 13.2 | Other | 13.4 | 11.3 |
| Elixhauser Comorbidities | Alcohol Use Disorder | 3.1 | Alcohol Use Disorder | 4.6 | 1.7 |
|  | Drug Use Disorder | 1.9 | Drug Use Disorder | 7.2 | 1.6 |
|  | Diabetes | 23.9 | Diabetes | 43.3 | 32.3 |
| Other comorbidities | SMI* | 13.6 | SMI* | 34.2 | 20.9 |
|  | Anxiety Disorders | 8.5 | Anxiety Disorders | 19.4 | 11.0 |
|  | Bipolar Disorders | 0.9 | Bipolar Disorders | 6.3 | 1.2 |
|  | Depressive Disorders | 7.7 | Depressive Disorders | 21.3 | 13.9 |
|  | Dissociative Disorders | 0.0 | Dissociative Disorders | 0.0 | 0.0 |
|  | Obsessive-Compulsive Disorders | 0.1 | Obsessive-Compulsive Disorders | 0.3 | 0.1 |
|  | Other Mood Disorders | 0.3 | Other Mood Disorders | 0.7 | 0.4 |
|  | Personality Disorders | 0.1 | Personality Disorders | 0.8 | 0.1 |
|  | Psychotic Disorders | 0.3 | Psychotic Disorders | 3.9 | 1.4 |
|  | Trauma-Related Disorders | 0.6 | Trauma-Related Disorders | 2.8 | 0.4 |

Abbreviations: Com=Commercial; MA=Medicare Advantage PI=Pacific Islander; SMI=serious mental illness.

*Serious mental illness (SMI) codes came from an Arizona block grant for serious emotional disturbance patients. The individual conditions that constitute SMI appear below SMI in the table. To qualify as having a condition, patients had to have at least one inpatient claim with a relevant diagnosis, or two outpatient claims at least two days apart with a relevant diagnosis in six months prior to January 2020 (July-December 2019).

Note: The numbers represent percentage of the cohort with that characteristic.

Table 2: Difference-in-Differences IRR, and Telemedicine Use for Select Specialties Among the Heart Failure Cohort April 2020-January 2022

|  |  | Jan ‘20 | Apr ‘20 | | Jul ‘20 | | Jul ‘21 | | Jan ‘22 | |
| --- | --- | --- | --- | --- | --- | --- | --- | --- | --- | --- |
| Insurance | Cohort | Rate per  100K PMs | DiD | Tel. | DiD | Tel. | DiD | Tel. | DiD | Tel. |
| Family Practice | | | | | | | | | | |
| Commercial | Heart Failure | 24,229 | 0.76* | 32% | 0.97 | 13% | 0.94† | 5% | 0.99 | 7% |
| MA <65 | Heart Failure | 61,371 | 0.84* | 24% | 1.01 | 11% | 0.89* | 5% | 0.87* | 9% |
| MA 65+ | Heart Failure | 56,117 | 0.78* | 21% | 0.96* | 10% | 0.84* | 4% | 0.81* | 7% |
| Pain Medicine | | | | | | | | | | |
| Commercial | Heart Failure | 2,893 | 0.92 | 32% | 1.09 | 14% | 1.07 | 3% | 1.00 | 8% |
| MA <65 | Heart Failure | 8,252 | 0.94 | 43% | 1.07 | 19% | 0.99 | 7% | 0.87† | 10% |
| MA 65+ | Heart Failure | 3,595 | 0.78* | 38% | 1.01 | 15% | 1.13* | 5% | 1.15* | 7% |
| Mental Health | | | | | | | | | | |
| Commercial | Heart Failure | 6,994 | 0.83* | 46% | 1.08 | 40% | 1.09‡ | 33% | 1.10‡ | 34% |
| MA <65 | Heart Failure | 14,849 | 0.96 | 43% | 1.03 | 36% | 1.02 | 25% | 1.08‡ | 28% |
| MA 65+ | Heart Failure | 8,169 | 0.82* | 31% | 0.98 | 26% | 0.90* | 16% | 0.89* | 18% |

| Abbreviations: DiD=Difference-in-differences; MA=Medicare Advantage; Tele.=telemedicine.  * p<0.001, † p<0.01, ‡ p<0.05 |
| --- |

Table 3: Rate and Difference-in-differences IRR for Emergency Medicine, Physical Therapy, and New Office Visits BETOS Categories Among the Heart Failure Cohort April 2020-January 2022

|  |  | Jan ‘20 | Apr ‘20 | Jul ‘20 | Jul ‘21 | Jan ‘22 |
| --- | --- | --- | --- | --- | --- | --- |
| Insurance | Cohort | DiD | DiD | DiD | DiD | DiD |
| Emergency Medicine | | | | | | |
| Commercial | Heart Failure | 9,326 | 0.57* | 0.83* | 0.88* | 0.95 |
| MA <65 | Heart Failure | 23,526 | 0.65* | 0.89* | 0.97 | 0.95‡ |
| MA 65+ | Heart Failure | 13,476 | 0.59* | 0.84* | 0.97† | 0.99 |
| Physical Therapy | | | | | | |
| Commercial | Heart Failure | 13,011 | 0.46* | 0.90* | 1.03 | 0.84* |
| MA <65 | Heart Failure | 22,381 | 0.70* | 1.01 | 0.90* | 0.77* |
| MA 65+ | Heart Failure | 34,207 | 0.75* | 1.05* | 0.98‡ | 0.81* |
| New Office Visits | | | | | | |
| Commercial | Heart Failure | 10,918 | 0.52* | 0.91† | 0.97 | 0.90† |
| MA <65 | Heart Failure | 11,511 | 0.51* | 0.94 | 0.93 | 0.85* |
| MA 65+ | Heart Failure | 9,747 | 0.40* | 0.92* | 1.04† | 0.91* |

| Abbreviations: DiD=difference-in-differences; IRR=incident rate ratio; MA=Medicare Advantage.  * p<0.001, † p<0.01, ‡ p<0.05 |
| --- |
