# Supplementary figures and images for: Health Services Usage in Patients Receiving Buprenorphine for Opioid Use Disorder or Long-Term Opioid Therapy for Chronic Pain: Retrospective Cohort Study
Source: JMIR Form Res. 2025 Jun 19;9:e66596. doi: 10.2196/66596 (PMC12226777; doi:10.2196/66596)

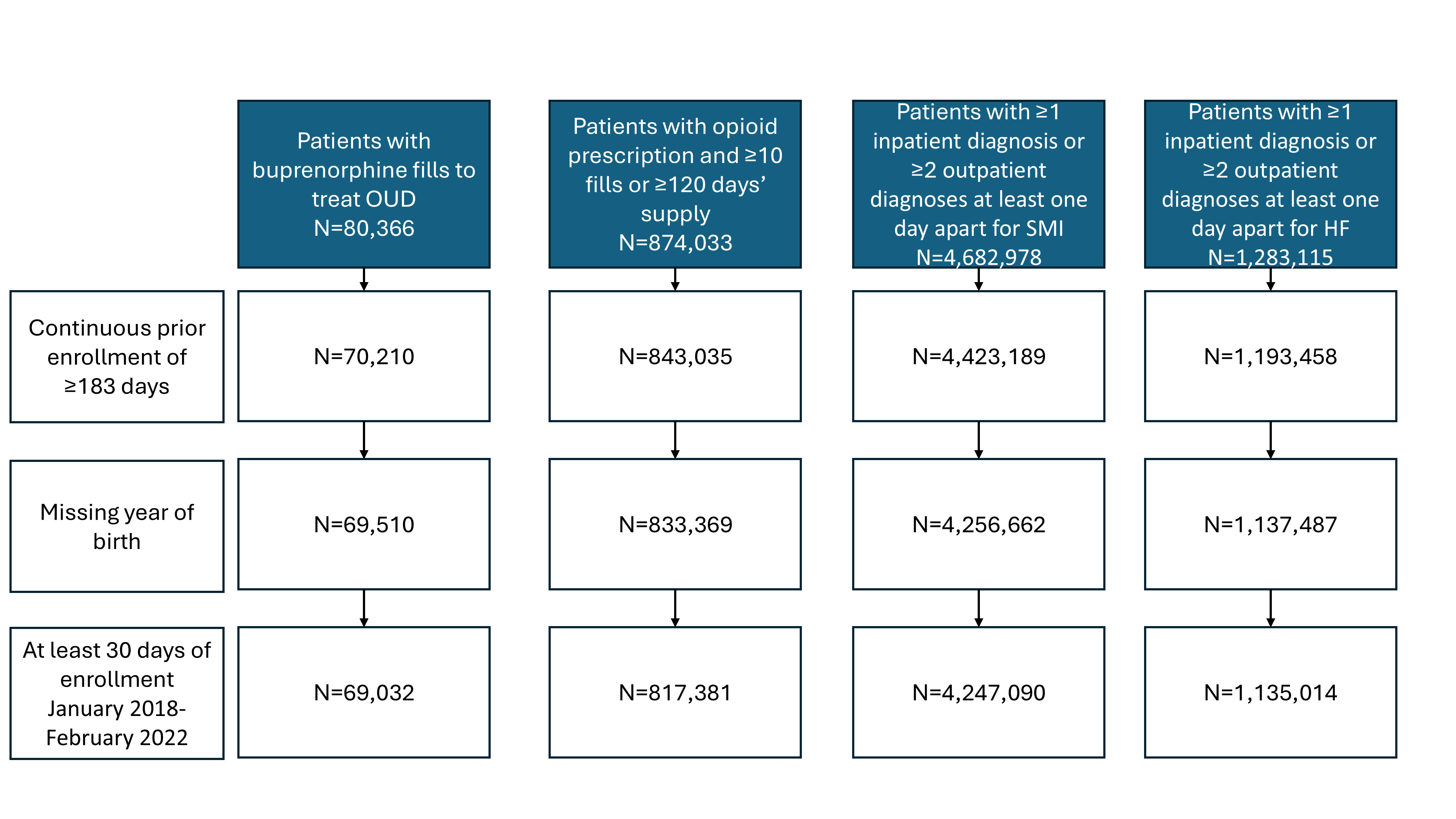

Supplement: Multimedia Appendix 3 [file formative_v9i1e66596_app3.png]

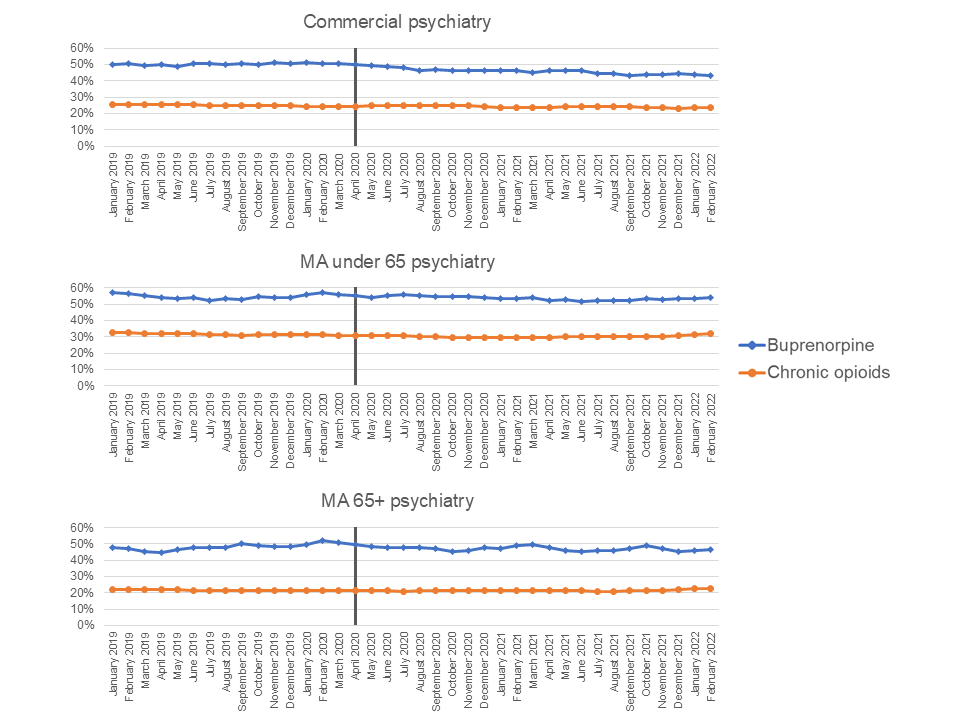

Supplement: Multimedia Appendix 5 [file formative_v9i1e66596_app5.png]
