# Supplementary material for: Health Services Usage in Patients Receiving Buprenorphine for Opioid Use Disorder or Long-Term Opioid Therapy for Chronic Pain: Retrospective Cohort Study
Source: JMIR Form Res. 2025 Jun 19;9:e66596. doi: 10.2196/66596 (PMC12226777; doi:10.2196/66596)
Supplement: Multimedia Appendix 4 [file formative_v9i1e66596_app4.docx]

## Appendix 2: Additional Results for Specialty Utilization

Table 1: Difference-in-differences IRR for Change in Utilization and Telemedicine Use Across Seven Specialty Categories in the Buprenorphine, Chronic Opioids, and Serious Mental Illness Cohorts Pre vs. Post COVID-19 April 2020 and January 2022

|  | Jan ‘20 | Apr ‘20 | | Jan ‘22 | |
| --- | --- | --- | --- | --- | --- |
| Cohort | Rate per  100K PMs | DiD | Tel. | DiD | Tel. |
| Cardiology | | | | | |
| Buprenorphine | 3,899.4 | 0.78† | 20% | 1.09 | 2% |
| Chronic Opioids | 11,283.1 | 0.64* | 20% | 0.97† | 2% |
| SMI | 10,370.3 | 0.65* | 19% | 0.88* | 2% |
| Emergency Medicine | | | | | |
| Buprenorphine | 5,918.3 | 0.60* | 6% | 1.09 | 2% |
| Chronic Opioids | 7,612.5 | 0.61* | 2% | 1.05* | 1% |
| SMI | 7,927.6 | 0.62* | 2% | 0.97* | 1% |
| Family Practice | | | | | |
| Buprenorphine | 30,630.0 | 0.92† | 36% | 1.07‡ | 14% |
| Chronic Opioids | 35,099.9 | 0.82* | 30% | 1.00 | 10% |
| SMI | 32,907.4 | 0.80* | 30% | 0.87* | 11% |
| Mental Health | | | | | |
| Buprenorphine | 32,527.8 | 0.99 | 45% | 1.05 | 35% |
| Chronic Opioids | 7,168.1 | 0.92* | 48% | 1.21* | 34% |
| SMI | 55,688.8 | 1.01* | 50% | 1.10* | 43% |
| Oncology | | | | | |
| Buprenorphine | 992.2 | - | 14% | - | 9% |
| Chronic Opioids | 5,620.9 | 0.81* | 12% | 0.92* | 4% |
| SMI | 3,864.3 | 0.77* | 15% | 0.81* | 5% |
| Orthopedics | | | | | |
| Buprenorphine | 2,740.0 | 0.50* | 9% | 1.05 | 2% |
| Chronic Opioids | 7,122.5 | 0.52* | 14% | 0.99 | 2% |
| SMI | 5,225.6 | 0.48* | 13% | 0.85* | 1% |
| Pain Medicine | | | | | |
| Buprenorphine | 6,247.1 | 1.08 | 49% | 0.95 | 15% |
| Chronic Opioids | 15,815.7 | 0.99 | 51% | 1.08* | 12% |
| SMI | 3,148.4 | 0.87* | 48% | 1.03‡ | 11% |

| Abbreviations: DiD=Difference-in-differences; Tele.=telemedicine. * p<0.001, † p<0.01, ‡ p<0.05. The IRR for oncology in the buprenorphine cohort is not displayed because the rate is below 1,000 person-days of service per 100,000 person-months. |
| --- |

Table 2: Difference-in-differences IRR for Change in Utilization and Telemedicine Use Across Seven Specialty Categories in the Buprenorphine, Chronic Opioids, and Serious Mental Illness Cohorts Pre vs. Post COVID-19 April 2020-January 2022

| Cardiology | | | | | | | | | | |
| --- | --- | --- | --- | --- | --- | --- | --- | --- | --- | --- |
|  |  | Jan ‘20 | Apr ‘20 | | Jul ‘20 | | Jul ‘21 | | Jan ‘22 | |
| Insurance | Cohort | Rate | DiD | Tel. | DiD | Tel. | DiD | Tel. | DiD | Tel. |
| Commercial | Bup. | 1,565.1 | 0.83 | 22% | 1.23 | 6% | 1.19 | 1% | 1.01 | 3% |
|  | CO | 4,523.1 | 0.60* | 22% | 0.94 | 6% | 0.96 | 1% | 0.91‡ | 2% |
|  | SMI | 3,132.6 | 0.61* | 22% | 0.99 | 7% | 0.97‡ | 2% | 0.84* | 3% |
| MA<65 | Bup. | 6,602.7 | 0.72‡ | 16% | 0.98 | 5% | 1.02 | 1% | 0.98 | 2% |
|  | CO | 10,958.1 | 0.69* | 19% | 1.01 | 6% | 1.08* | 2% | 0.88* | 3% |
|  | SMI | 14,360.3 | 0.72* | 16% | 0.96‡ | 5% | 0.99 | 1% | 0.83* | 2% |
| MA 65+ | Bup. | 11,568.5 | 0.75‡ | 22% | 1.12 | 6% | 1.27 | 1% | 0.73 | 2% |
|  | CO | 14,003.2 | 0.62* | 20% | 0.99 | 5% | 1.12* | 1% | 1.01 | 2% |
|  | SMI | 20,397.8 | 0.63* | 18% | 0.95* | 5% | 1.02‡ | 1% | 0.91* | 2% |
| Emergency Medicine | | | | | | | | | | |
|  |  | Jan ‘20 | Apr ‘20 | | Jul ‘20 | | Jul ‘21 | | Jan ‘22 | |
| Insurance | Cohort | Rate | DiD | Tel. | DiD | Tel. | DiD | Tel. | DiD | Tel. |
| Commercial | Bup. | 3,877.1 | 0.54* | 12% | 0.88 | 6% | 0.99 | 3% | 1.18 | 4% |
|  | CO | 5,553.1 | 0.60* | 3% | 0.83* | 1% | 0.90† | 0% | 1.06 | 1% |
|  | SMI | 4,323.9 | 0.57* | 4% | 0.96† | 2% | 0.93* | 1% | 0.97 | 1% |
| MA<65 | Bup. | 9,685.6 | 0.62* | 3% | 0.88 | 1% | 0.91 | 1% | 0.93 | 1% |
|  | CO | 10,153.1 | 0.60* | 1% | 0.82* | 1% | 0.98 | 0% | 0.96 | 1% |
|  | SMI | 15,994.3 | 0.65* | 1% | 0.87* | 0% | 0.93* | 0% | 0.87* | 1% |
| MA 65+ | Bup. | 9,691.2 | 0.64† | 1% | 1.14 | 2% | 1.19 | 1% | 0.71 | 1% |
|  | CO | 7,096.8 | 0.62* | 1% | 0.92* | 1% | 1.16* | 0% | 1.08* | 1% |
|  | SMI | 10,568.3 | 0.63* | 1% | 0.88* | 0% | 1.04* | 0% | 1.01 | 1% |
| Oncology | | | | | | | | | | |
|  |  | Jan ‘20 | Apr ‘20 | | Jul ‘20 | | Jul ‘21 | | Jan ‘22 | |
| Insurance | Cohort | Rate | DiD | Tel. | DiD | Tel. | DiD | Tel. | DiD | Tel. |
| Commercial | Bup. | 515.8 | 0.65 | 17% | 0.35* | 6% | 0.75 | 5% | 1.13 | 4% |
|  | CO | 5,148.7 | 0.94 | 10% | 1.02 | 6% | 1.02 | 3% | 0.92‡ | 4% |
|  | SMI | 1,951.6 | 0.83* | 13% | 0.99 | 7% | 0.87* | 3% | 0.75* | 4% |
| MA<65 | Bup. | 1,626.4 | 0.65 | 16% | 0.72 | 3% | 0.75 | 5% | 1.75‡ | 9% |
|  | CO | 4,939.2 | 0.81* | 14% | 0.91† | 7% | 0.97 | 2% | 0.83* | 5% |
|  | SMI | 4,583.6 | 0.79* | 15% | 0.90* | 7% | 0.90* | 3% | 0.82* | 5% |
| MA 65+ | Bup. | 2,384.7 | 2.54* | 11% | 1.86‡ | 9% | 2.43* | 7% | 1.48 | 6% |
|  | CO | 6,146.6 | 0.77* | 13% | 0.95‡ | 5% | 1.03 | 3% | 0.98 | 4% |
|  | SMI | 6,644.1 | 0.73* | 15% | 0.99 | 7% | 0.97 | 3% | 0.84* | 5% |
| Orthopedics | | | | | | | | | | |
|  |  | Jan ‘20 | Apr ‘20 | | Jul ‘20 | | Jul ‘21 | | Jan ‘22 | |
| Insurance | Cohort | Rate | DiD | Tel. | DiD | Tel. | DiD | Tel. | DiD | Tel. |
| Commercial | Bup. | 1,636.2 | 0.38* | 7% | 1.04 | 3% | 0.95 | 0% | 0.95 | 0% |
|  | CO | 6,701.5 | 0.57* | 16% | 0.92† | 4% | 0.96 | 2% | 0.95 | 2% |
|  | SMI | 3,083.7 | 0.52* | 17% | 0.92 | 3% | 0.92 | 1% | 0.81* | 1% |
| MA<65 | Bup. | 4,199.5 | 0.48* | 7% | 0.93* | 3% | 0.89* | 0% | 0.78* | 2% |
|  | CO | 7,616.1 | 0.54* | 14% | 1.18 | 3% | 0.99 | 1% | 1.05 | 2% |
|  | SMI | 7,379.2 | 0.59* | 12% | 0.99 | 2% | 0.99 | 1% | 0.90* | 2% |
| MA 65+ | Bup. | 5,987.2 | 0.51* | 14% | 0.84* | 3% | 0.82* | 2% | 0.79* | 5% |
|  | CO | 7,030.1 | 0.56* | 13% | 0.98 | 2% | 0.95‡ | 1% | 0.87* | 1% |
|  | SMI | 7,816.7 | 0.60‡ | 11% | 1.05 | 2% | 0.98 | 1% | 0.85 | 1% |
| Commercial | Bup. | 1,636.2 | 0.47* | 7% | 0.92* | 3% | 1.03 | 0% | 1.02 | 0% |
|  | CO | 6,701.5 | 0.44* | 16% | 0.91* | 4% | 0.98 | 2% | 0.84* | 2% |
|  | SMI | 3,083.7 | 0.44* | 17% | 0.88* | 3% | 0.95* | 1% | 0.88* | 1% |
